# Factors associated with the access and continuum of vaccination services among children aged 12–23 months in the emerging regions of Ethiopia: evidence from the 2016 Ethiopian demography and health survey

**DOI:** 10.1186/s13052-020-0793-9

**Published:** 2020-03-04

**Authors:** Ayal Debie, Ayenew Molla Lakew

**Affiliations:** 1 Department of Health Systems and Health Policy, Institute of Public Health, University of Gondar, P.O. Box: 196, Gondar, Ethiopia; 2 Department of Epidemiology and Biostatistics, Institute of Public Health, University of Gondar, Gondar, Ethiopia

**Keywords:** Access, Continuum, Vaccination, Children, Emerging region, Ethiopia

## Abstract

**Background:**

Child vaccination is an instrument for saving millions of lives. Only one in twenty children has access to childhood vaccination in hard to reach areas in developing countries. Although studies have been done on childhood vaccination, factors associated with access and continuum were not considered in Ethiopia. Therefore, this study aimed to identify the factors associated with the access and continuum of childhood vaccination in the emerging regions of Ethiopia based on the 2016 EDHS datasets.

**Methods:**

The two-stage stratified sampling technique was used for the survey carried out on 642 mothers of children aged 12–23 months. Access is the provision of services in shorter waiting times and flexibly at all times and alternative methods of communication. Accordingly, continuum of care reflects the extent to which a series of discrete health care events are being experienced by people coherently and interconnected over time. As a result, access and continuum of childhood vaccination are determined using pentavalent-1 and measles vaccination status of children, respectively. A binary logistic regression model was fitted to identify the factors associated with access and continuum of the vaccination.

**Results:**

Overall, 25.1% of children aged 12–23 months received all of the recommended childhood vaccines. Sixty-two percent of children accessed and 46.9% had continuum of childhood vaccination in the emerging regions of Ethiopia. Pentavalent_1 to 3 and BCG to measles dropout rates were 33.42 and 17.53%, respectively. Mothers’ formal education (AOR = 1.99; 95%CI: 1.20, 3.31), ANC (AOR = 4.13; 95%CI: 2.75,6.19), health facility delivery of last birth (AOR = 1.58; 95%CI: 1.19, 2.82), rich wealth (AOR = 1.57; 95%CI: 1.19, 3.14) and average child birth weight (AOR = 1.67; 95%CI: 1.03, 2.72) were positively associated with childhood access to vaccination. On the other hand, mothers’ ANC attendance (AOR = 3.68; 95%CI: 2.48, 5.47) and rich wealth (AOR = 2.07; 95%CI: 1.15, 3.71) were positively associated with the continuum of the services. On the contrary, children with rural resident mothers (AOR = 0.33; 95%CI: 0.14, 0.76) and small birth weight (AOR = 0.51; 95%CI: 0.33, 0.81) were negatively associated to the access and continuum of childhood vaccination, respectively.

**Conclusion:**

Childhood vaccination status was low in the emerging regions of Ethiopia. Variables such as maternal education, birth weight of children, ANC, health facility delivery and wealth were associated with the access and continuum of the vaccination. Therefore, empowering women with education and strengthening maternal healthcare services might enhance childhood vaccination. In addition, the government needs to design a compensation mechanism for the cost relating to childhood vaccination to improve the access and continuum of the service.

## Background

The Expanded Programme for Immunization (EPI) was initiated by the World Health Organization (WHO) in 1974 [1]. The WHO advocated the availability of immunization for all children of the world by the year 1990 which was a vital step towards the achievement of health for all by the year 2000 [2]. Childhood vaccination is an instrument of saving millions of lives, but about 19.4 million children under the age of 1 year have not received basic vaccines [3]. Immunization prevents 2–3 million deaths every year, and the uptake of new vaccines has been increasing from time to time [3].

Childhood vaccination is effective in protecting children against vaccine-preventable diseases in low- and middle-income countries [4, 5]. In Sub-Saharan Africa and hard to reach (remote areas) of developing countries, only 50% of children and one in twenty had access to childhood vaccination, respectively [6]. In 2018, about 86%(116.3 million) of infants worldwide received three doses of DTP-3 /Penta-3 vaccine [3].

Childhood vaccination is one of the national child survival strategies planned to reach 90% of the measles vaccination coverage in 2010 [7]. In 2015, it was also one of the key intervention strategies to achieve the Health Sector Transformation Plan (HSTP) of Ethiopia [8]. The 2011 EDHS revealed that 66, 56, 64 and 24% of children received BCG, measles, pentavalent-1 and all the recommended vaccines, respectively [9], while the 2016 EDHS showed 69, 54, and 73% of children had BCG, measles, and pentavalent-1 vaccines, accordingly. In addition, 39 and 16% of children were fully vaccinated and never received any childhood vaccinations, respectively [10]. According to the 2016 EDHS report, childhood vaccination was low in the emerging regions of Ethiopia [10]. The vaccination status of children is used to monitor the performance of vaccination services at local, national and international levels to design strategies for the eradication, elimination, and control of vaccine-preventable diseases [11–13].

Although a few studies so far been done in different parts of Ethiopia, the social determinants of vaccination service utilization remain poorly analyzed, particularly in the emerging regions. Therefore, this study aimed to assess factors associated with the access and continuum of childhood vaccination service utilization among children aged 12–23 months in the emerging regions of Ethiopia based on the 2016 EDHS datasets.

## Methods

### Study settings

The 2016 Ethiopian Demographic and Health Survey (EDHS) was conducted in the nine national regional states and the two city administrations. The regions classified as emerging, Afar, Benishangul-Gumz, Gambella and Somali, are characterized by scattered pastoralists and semi-pastoralist societies suffering from extreme poverty. Absence of clear and detailed regulations, basic infrastructures and services are also their common chrematistics [14, 15]. On the other hand, developed regions such as Amhara, Oromia, Southern Nations, Nationalities and Peoples (SNNP), Tigray and the city administrations, such as Addis Ababa, Dire Dawa and Harari regions are relatively more densely populated [15]. Ethiopia is one of the Sub-Saharan countries found in the horn of Africa with a total population of 73.5 million. The total population of the four emerging regions was 6926, 933, with the largest in Somali (4,445,219) and the least in Gambella (307,096). Similarly, the total number of children 0–4 years was 449,699 in Somali and 42,044 in Gambella [16].

### Sampling design

The sampling frame used for the 2016 EDHS was the 2007 Population and Housing Census (PHC) of the Central Statistical Agency report of Ethiopia [16]. The sample for the 2016 EDHS was designed to provide estimates of the key indicators of the country as a whole, separately for urban and rural areas, and for each of the nine regions and the two city administrations. The sample was stratified and selected in two stages and each region was stratified into urban and rural areas. Samples of the Enumeration Areas (EAs) were selected independently in each stratum of two stages. Implicit stratification and proportional allocation were used at each lower administrative level.

In the first stage, EAs in urban and rural areas were selected with probability proportional to the EA size (based on the 2007 PHC) and with independent selection in each sampling stratum. A household listing operation was carried out in all of the selected EAs in 2015. The resulting lists of households served as sampling frames for the selection of households in the second stage. Segmentation was done for some of the selected EAs with large households, and only one segment was selected for the survey with a probability proportional to size. Household listing was conducted only in the selected segment, that is, the 2016 EDHS cluster was either an EA or a segment of an EA. In the second stage, the selection of the households per cluster was done using systematic sampling technique. In this study, the 2016 Ethiopian demographic and health survey childhood datasets of the four emerging regions, namely Afar, Benishangul-Gumz, Gambella and Somali were used for analysis.

All women aged 15–49 years and permanently lived in the area and slept in the selected households the night before the surveys were eligible [10]. Children 12–23 months are the source population and the study included 642 mothers and their children aged 12–23 months and data on both were extracted from the 2016 EDHS datasets. Potential independent variables such as socio-demographic, economic, fertility history and health service utilization were also extracted and further recoding of the selected variables was done to match and compare with other similar studies.

### Measurement and variables

Access and continuum of childhood vaccination were the dependent variables of the study. Socio-demographic characteristics (age, residence, religion, and marital status), and obstetric history of the women, like places of delivery, birth order, antenatal care, postnatal check-ups in 2 months after birth, number of live children, sex of children, and marital status were the independent variables. Vaccination refers to the administration of antigenic material (a vaccine) to stimulate an individual’s immune system to develop adaptive immunity to a pathogen [17] and its coverage is defined as the proportion of a given population that has been vaccinated in a given time period [18]. A fully vaccinated child was expected to receive one dose of BCG, three doses of pentavalent, Pneumococcal Conjugate (PCV), Oral Polio Vaccines (OPV), two doses of Rotavirus and one dose of measles vaccines [19]. Each vaccine had five response categories, namely “no”, “vaccination date on the card”, “reported by mothers”, “vaccination marked on card” and “do not know”. The vaccination status of children was recoded as 0 and 1 for each antigen. “No” responses were recoded as “0” and labeled “not received the vaccine”, while the other responses “vaccination date on card, reported by mothers, vaccination marked on card” were recoded together as “1” and labeled “received the vaccine”. Besides, “do not know responses” were excluded from analysis. As a result, the vaccination status of children was recoded as “0” for “not vaccinated” and “1” for “vaccinated” for each antigen on the basis of the reports of women and information on the child vaccination card. Accordingly, access and the continuum of childhood vaccination were recoded as “0” for “no” and “1” for “yes” for each child. Access is the provision of services in shorter waiting times, more flexibly, electronically, by telephone or alternative methods of communication [20]. As a result, access to childhood vaccination was determined based on pentavalent-1 vaccination status of children. Continuum of care reflects the extent to which a series of discrete health care events are being experienced by people coherently and interconnected over time [20]. Thus, continuum of childhood vaccination was measured by measles vaccination status of children.

### Data management and statistical analyses

The extracted EDHS data included socio-demographic characteristics of the women, obstetric history and service utilization child-specific information for all births in the past 5 years of women in the reproductive age group. The 2016 EDHS collected information on childhood vaccination status from vaccination cards and women’s verbal reports. The interviewer copied the vaccination dates directly into the questionnaire if the cards were available. However, the interviewer asked respondents to recall the vaccines given to their children when there were no vaccination cards. The cleaned and recoded data were analyzed using STATA version 14. Descriptive statistics such as means, medians, SDs, frequencies and proportions of variables were presented using graphs, texts, and tables. Bivariable and multivariable logistic regression analyses were conducted to identify factors associated with access, utilization, and continuity of vaccination. Variables with *p*-values < 0.2 [21, 22] during the bivariable analyses were fitted into the multivariable logistic regression analyses. However, *p*-value of 0.2 did not mean that there was a 20% chance that the null hypothesis was correct [22, 23], rather variables with *p*-value of < 0.2 during the bivariable analyses might have a chance to be significantly associated with the outcome variable during the multivariable regression analyses. Adjusted Odds Ratio (AOR) and 95% Confidence Interval (CI) with *p*-value < 0.05 were used to identify variables associated with the outcome variables.

## Results

### Socio-demographic and economic characteristics

A total of 642 mothers/caregivers of children 12–23 months of age were included in these analyses. The mean age of the participants was 27.86 (+ 6.31SD) years, and the median age was 28 years with an Inter Quartile Range (IQR) of 8 years. Furthermore, more than half of the participants were in the age range of 25–34 years; over two-thirds (68.2%) were Muslims; more than 80 % (83.2%) lived in rural areas. Only 8.9 and 19.5% of the respondents and their husbands had secondary and above education, respectively. Similarly, 5.1% of the respondents and 13.2% of their husbands had professional jobs; 93.3% were married (Table 1).

**Table 1:** Socio-demographic and economic characteristics of participants in emerging regions of Ethiopia, 2016

Variables	Frequency	Percent (%)
**Age of the respondents in years**		
**15–24**	198	30.8
**25–34**	329	51.2
**≥ 35**	115	18.0
**Religion of the participants**		
**Orthodox**	61	9.5
**Protestant**	116	18.1
**Muslim**	438	68.2
**Others**	27	4.2
**Region of the respondents**		
**Afar**	162	25.2
**Somali**	196	30.5
**Benshangul_Gumuz**	152	23.7
**Gambella**	132	20.6
**Residence of the participants**		
**Urban**	108	16.8
**Rural**	534	83.2
**Educational status of the respondents**		
**No education**	458	71.3
**Primary**	127	19.8
**Secondary and above**	57	8.9
**Sex of household head**		
**Male**	426	66.4
**Female**	216	33.6
**Husband education**		
**No**	408	63.5
**Primary**	109	17.0
**Secondary and above**	125	19.5
**Current marital status of the respondents**		
**Unmarried**	43	6.7
**Married**	599	93.3
**Husband occupation**		
**No work**	93	14.5
**Professional work**	85	13.2
**Agricultural**	293	45.6
**Others**	171	26.6
**Occupation of the respondents**		
**No work**	418	65.1
**Professional work**	33	5.1
**Agricultural**	128	19.9
**Others**	63	9.8

### Obstetric related characteristics of participants

More than half (57.5%) of the women received antenatal care services. Accordingly, 44 and 65.6% of the ANC users had four and/or more visits and started the service at the second trimester, respectively. Similarly, only 27.6 and 8.1% of the women had health facility delivery and PNC in 2 months of postpartum period, correspondingly. Nearly 27, 62 and 7% of the participants had ≥6, ≥3 and history of terminated pregnancy, respectively (Table 2).

**Table 2:** Obstetric related characteristics of participants in emerging regions of Ethiopia, 2016

Variables	Frequency	Percent (%)
**ANC**		
**No**	273	42.5
**Yes**	369	57.5
**ANC number of visits (*n*** **= 369)**		
**1**	39	10.6
**2–3**	168	45.5
**≥ 4**	162	43.9
**First ANC visit started(*n*** **= 369)**		
**< 3 months**	109	29.5
**3–7 months**	242	65.6
**> 7 months**	18	4.9
**Place of delivery**		
**At home**	465	72.4
**At health facility**	177	27.6
**PNC within 2 months**		
**No**	590	91.9
**Yes**	52	8.1
**Place of PNC(*n*** **= 52)**		
**At home**	15	28.8
**At health facility**	37	71.2
**PNC performed by(*n*** **= 52)**		
**Doctor**	6	11.5
**Nurse**	28	53.8
**Midwife**	3	5.8
**HEW**	15	28.9
**Parity**		
**1**	113	17.6
**2–5**	358	55.8
**≥ 6**	171	26.6
**Number of living children**		
**1**	119	18.5
**2**	125	19.5
**≥ 3**	398	62.0
**Ever had history of terminated pregnancy**		
**No**	598	93.1
**Yes**	44	6.9

### Source of information, wealth status, and child characteristics

Of the children, 55.6% were female and60.6% of the caregivers were in the poorest wealth status. The mean and median age of the children were 16.29 (+ 3.29SD) months and 16 months with the IQR of 6 months, respectively. About 45% of the children were in the age range of 12–15 months. More than three-fourths (76.5%) of participants had no mobile phones, and almost 85% did not listen radio programs. Nearly 40 and 90% of children had average birth weights and wanted by their families, respectively (Table 3).

**Table 3:** Source of information, wealth status of participants and child characteristics in emerging regions of Ethiopia, 2016

Variables	Frequency	Percent (%)
**Sex of child**		
**Male**	357	55.6
**Female**	285	44.4
**Child age in months**		
**12–15**	288	44.9
**16–19**	224	34.9
**20–23**	130	20.2
**Wealth status**		
**Poorest**	389	60.6
**Poor**	81	12.6
**Middle**	43	6.7
**Rich**	53	8.3
**Richest**	76	11.8
**Own a mobile phone**		
**No**	491	76.5
**Yes**	151	23.5
**Reading newspaper or magazine**		
**No**	620	96.6
**Yes**	22	3.4
**Listening radio**		
**No**	547	85.2
**Yes**	95	14.8
**Watching TV**		
**No**	569	88.6
**Yes**	73	11.4
**Wanted last child**		
**No**	71	11.1
**Yes**	571	88.9
**Child size at birth**		
**Large**	175	27.3
**Average**	256	39.9
**Small**	211	32.8
**Birth order of the child**		
**1**	113	17.6
**2–5**	358	55.8
**≥ 6**	171	26.6
**Preceding birth interval in months**		
**9–23**	133	20.7
**24–35**	188	29.3
**> 35**	321	50.0

### Vaccination status of children

**Fig. 1:**
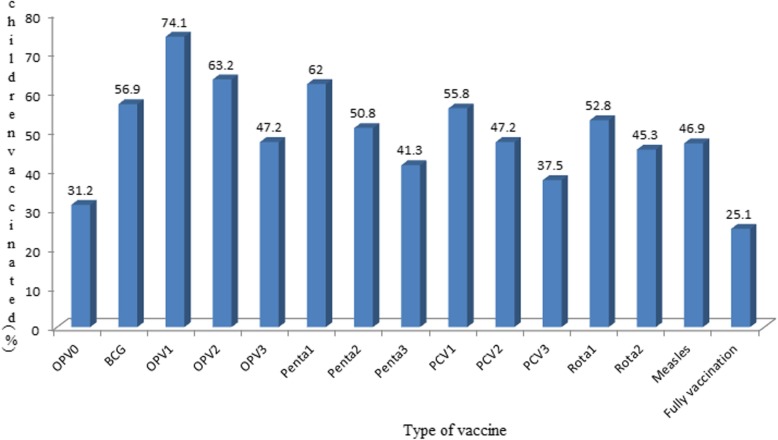
Vaccination status of children aged 12–23 months for each antigen in emerging regions of Ethiopia, 2016

The vaccination status of children 12–23 months of age was analyzed for each antigen in the emerging regions of Ethiopia. Overall, 25.1% (95%CI: 21.80, 28.30) of the children were fully vaccinated in the region. About 57 and 47%of the children received BCG and measles vaccines, accordingly. Seventy-four-point-four and forty-seven-point 2 % of children received OPV 1 and 3, respectively. About 62.0, 41.3, 55.8, 37.5, 52.8, and 45.3% had received Pentavalent 1, 2, PCV 1, 2, Rota 1 and 2, correspondingly. Furthermore, pentavalent and BCG to measles dropout rates were 33.4 and 17.5%, respectively. As a result, access to childhood vaccination was 62% (95%CI: 58.40, 65.70) and the continuum of vaccination 46.9% with (95%CI: 43.30, 50.80) (Fig. 1).

### Factors associated with access to vaccination

The binary logistic regression model showed that educational status of the mothers, residence, weight of children, ANC follow ups and places of birth were significantly associated with access to childhood vaccination. Mothers who lived in rural areas were 67% less likely (AOR = 0.33; 95%CI: 0.14,0.76) access to their children vaccination compared with that of mothers lived in urban areas. Accordingly, participants who had formal education were 1.99 times (AOR = 1.99; 95%CI: 1.20, 3.31) more likely to get access to vaccination for their children compared with women who had no formal education. On the other hand, children who had average birth weights were1.67 times (AOR = 1.67; 95%CI: 1.03, 2.72) more likely to get access to vaccination compared with children who had more birth weights. The odds of childhood access to vaccination among women who had ANC follow ups were 4.13 times (AOR = 4.13; 95%CI: 2.75,6.19) higher than those of their counterparts. Women who gave their last birth at health facilities were 1.58 times (AOR = 1.58; 95%CI: 1.19, 2.82) more likely to have chances to access child vaccinations compared with women who gave birth at home. Similarly, women with middle (AOR = 2.79; 95%CI: 1.09, 7.14) and rich wealth (AOR = 1.57; 95%CI: 1.19, 3.14) had 2.79 and 1.57-times higher access to childhood vaccination compared with women in poor wealth status (Table 4).

**Table 4:** Factors associated with access for childhood vaccination in emerging regions of Ethiopia, 2016

Variables	Access to vaccination		COR (95%CI)	AOR (95% CI)	*P*-value
	Yes	No			
**ANC**					
**No**	100	173	1	1	
**Yes**	298	71	7.26(5.08,10.38)	4.13(2.75,6.19) *	0.0001
**Own a mobile phone**					
**No**	282	209	1	1	
**Yes**	116	35	2.46(1.62,3.73)	1.29(0.75, 2.19)	0.357
**Wanted last child**					
**No**	51	20	1	1	
**Yes**	347	224	0.61(0.35,1.05)	0.74(0.38, 1.44)	0.382
**History of terminated pregnancy**					
**No**	367	231	1	1	
**Yes**	31	13	1.50(0.77,2.93)	1.58(0.71, 3.53)	0.266
**Listening radio**					
**No**	322	225	1	1	
**Yes**	76	19	2.80(1.64,4.75)	1.87(0.98,3.49)	0.052
**Place of delivery**					
**At home**	244	221	1	1	
**At health facility**	154	23	6.07(3.77,9.75)	1.58(1.19, 2.82) *	0.018
**Child size at birth**					
**Large**	112	63	1	1	
**Average**	182	74	1.38(0.92,2.09)	1.67(1.03, 2.72) *	0.039
**Small**	104	107	0.55(0.36,0.82)	0.63(0.39, 1.03)	0.064
**PNC within 2 months**					
**No**	353	237	1	1	
**Yes**	45	7	4.32(1.91,9.73)	1.63(0.64, 4.13)	0.306
**Wealth status**					
**Poor**	249	221	1	1	
**Middle**	37	6	5.85(3.40,10.05)	2.79(1.09, 7.14) *	0.032
**Rich**	112	17	5.47(2.27,13.21)	1.57(1.19, 3.14) *	0.012
**Education of respondents**					
**No formal education**	245	213	1	1	
**Formal education**	153	31	4.29(2.80,6.58)	1.99(1.20, 3.31) *	0.007
**Residence**					
**Urban**	98	10	1	1	
**Rural**	300	234	0.13(0.07,0.26)	0.33(0.14,0.76) *	0.010

*significant at *p*-value < 0.05, *COR* Crude Odds Ratio, *AOR* Adjusted Odds Ratio

### Factors associated with the continuum of vaccination

Potential predictor variables associated with the continuum of vaccination services among children 12–23 months of age were identified using the multivariable logistic regression analyses. Children who had less birth weights were 49% less likely (AOR = 0.51; 95%CI: 0.33, 0.81) to continue vaccination services compared with children who had more birth weights. The odds of the continuum of childhood vaccination among women who had ANC follow ups were 3.68 times (AOR = 3.68; 95%CI: 2.48, 5.47) higher than women who had no ANC. Women in rich wealth status were 2.07 times (AOR = 2.07; 95%CI: 1.15, 3.71) more likely to continue childhood vaccination compared with women in poor wealth status (Table 5).

**Table 5:** Factors associated with the continuum of childhood vaccination in emerging regions of Ethiopia, 2016

Variables	Continuum of vaccination		COR (95%CI)	AOR (95% CI)	*p*-value
	Yes	No			
**ANC**					
**No**	66	207	1	1	
**Yes**	235	134	5.50(3.88,7.80)	3.68(2.48, 5.47) *	0.0001
**Own a mobile phone**					
**No**	213	278	1	1	
**Yes**	88	63	1.82(1.26, 2.64)	1.06(0.75, 1.84)	0.815
**Parity**					
**1**	64	49	1	1	
**2–5**	177	181	0.75(0.49, 1.15)	0.84(0.53, 1.39)	0.482
**≥ 6**	60	111	0.41(0.25, 0.67)	0.58(0.32, 1.05)	0.074
**Place of delivery**					
**At home**	177	288	1	1	
**At health facility**	124	53	3.81(2.62, 5.52)	1.44(0.89, 2.31)	0.136
**History of terminated pregnancy**					
**No**	277	321	1	1	
**Yes**	24	20	1.39(0.75, 2.57)	1.39(0.67, 2.88)	0.369
**Child size at birth**					
**Large**	90	85	1	1	
**Average**	142	114	1.18(0.80,1.73)	1.34(0.86, 2.08)	0.192
**Small**	69	142	0.46(0.30,0.69)	0.51(0.33, 0.81) *	0.004
**PNC within 2 months**					
**No**	263	327	1	1	
**Yes**	38	14	3.38(1.79, 6.36)	1.75(0.87, 3.62)	0.121
**Wealth status**					
**Poor**	184	286	1	1	
**Middle**	23	20	1.79(0.96, 3.35)	1.08(0.45, 1.83)	0.954
**Rich**	94	35	4.18(2.72, 6.42)	2.07(1.15, 3.71) *	0.015
**Education of respondents**					
**No formal education**	178	280	1	1	
**Formal education**	123	61	3.17(2.21, 4.55)	1.41(0.90, 2.21)	0.138
**Sex of child**					
**Male**	162	195	0.87(0.64,1.19)	0.81(0.56, 1.16)	0.271
**Female**	139	146	1	1	
**Residence**					
**Urban**	76	32	1	1	
**Rural**	225	309	0.31(0.20,0.48)	0.90(0.49,1.65)	0.721

*significant at *p*-value < 0.05, *COR* Crude Odds Ratio, *AOR* Adjusted Odds Ratio

## Discussion

Ethiopia was committed to improve the access and continuum of vaccination to achieve full vaccination via designing health policies and strategies, such as the construction of health posts, training and deployment of health extension workers. The country has also made great gains in decreasing childhood and under-five mortality by two-thirds since 1990 and meeting its Millennium Development Goal target [24]. However, attaining the Sustainable Development Goal for reducing under-5 mortality from 59 deaths in 2015 [25] to 25 deaths per 1000 live births in 2030 [26] will require improved child health services through ensuring the access and continuum of childhood vaccination services. Despite access to childhood vaccination in the country, many children do not receive the vaccines.

As a result,25.1% (95%CI: 21.80, 28.30) of the children were fully vaccinated that does not lead in achieving the 2020 Health Sector Transformation Plan (HSTP). This finding was consistent with the 2011 EDHS report (24%) [9] and the Kenyan (22.6%) [27]. However, it was lower than those of studies done in Togo, Nigeria (63.7%) [28], Kwahu Afram Plains, Ghana (81.3%) [29], Jigjiga (36.6%) [30], Mecha (75.1%) [31], Ethiopia (38.3%) [32], Sekota Zuria (77.4%) [33], Areka town (75.4%) [34], Wonago (52.4%) [35], and Ambo (35.6%) [36]. However, it was higher than the results of studies in Amibara (8.3%) [37] and the 2005 EDHS report (20%) [38]. Accordingly, 62% (95%CI: 58.40, 65.70) of children aged 12–23 months of age had access to childhood vaccination. The finding was lower than those of studies done in Mecha (98.4%) [31], Jigjiga (73%) [30], Debre Markos (96.9%) [39], Wonago (99.0%) [35], Kwahu Afram Plains, Ghana (97.3%) [29], but higher than that of a study done in Ambo(36.9%) [36]. The continuum of childhood vaccination services in the current study was 46.9% (95%CI: 43.30, 50.80). The finding was lower than the results of studies done in Debre Markos (91.7%) [39], Ethiopia (55.7%) [40], Kwahu Afram Plains, Ghana (87.7%) [29], but higher than that of a study done in Ambo (29.9%) [36]. Additionally, pentavalent-1 to 3 and BCG to measles dropout rates in this study were 33.42 and 17.53%, respectively. The possible explanation for this variation might be the differences in study periods and designs. Another justification might be the current analyses was done in pastoral communities where the necessary information about childhood vaccination was not available. Another reasons could be the differences in health system from country to country. Furthermore, the number of vaccine types in the current assessment of childhood complete vaccination status is different from the previous ones, and this can result in variations in vaccination status of children.

Mothers who lived in rural areas negatively influenced the access to childhood vaccination compared with that of mothers lived in urban areas. This finding was supported by the studies done in Mecha [31], East Gojjam [41] and Jigjiga [30]. The possible justification might be that urban resident mothers might have better information and recognize the importance of vaccination.

Women who had formal education positively influenced access to childhood vaccination compared with non-educated ones. This finding was supported by those of studies done in Kenya [27], Jigjiga, Amibara, and Sekota Zuria [30, 32, 33, 37], Ethiopia, and Nigeria [42]. The possible justification might be that educated mothers might have better knowledge about vaccine-preventable diseases and recognize the importance of vaccination.

Women who received ANC during their last pregnancy positively influenced the access and continuum of the vaccination of children compared with mothers who had no ANC at all. This finding is consistent with other findings in Ethiopia [32, 33, 35, 43, 44], Nigeria [45], Uganda [46], and Kenya [47].

The access and continuum of childhood vaccination were higher among women who gave their last birth at health institutions compared with those delivered at home. This finding was also in line with those of studies conducted in Ethiopia [30–33, 48] and Kenya [47]. The higher chance of getting access, continuum and complete childhood vaccination of children born to women who had utilized ANC and/ or institutional delivery services could be related to their familiarity to the healthcare systems during their previous visits and health workers’ advice on the vaccination of children.

Women in the rich wealth status positively influenced the access and continuum of the vaccination status of children compared with their counterparts. This finding was consistent with those of other studies in Ethiopia [32, 40, 49], and Bangladesh [50]. The possible justification might be that rich households give more attention to their children. Additionally, they could not suffer from financial scarcity for transportation to get services.

Mothers who had low birth weight children negatively influenced childhood access and continuum of vaccination compared with mothers who had more birth weight children. The possible explanation might be that mothers who had low birth weight children could not take their child for vaccination sessions because they consider their children were not normal and for fear of vaccine side effects. As these mothers might focus on improving their children’s weight, they could not give attention to other child health issues.

This study has a number of limitations. It lacks the effect of vaccine management system and service delivery related factors, like logistics, knowledge of clients, attitude and trained human resources as predictors of childhood vaccination. Besides, it did not cover infrastructure and human resources that might have contributed to the low access and continuum of vaccination. Moreover, the study did not address the reasons for low access and continuum of vaccination and the immune status of vaccinated children.

## Conclusion

The overall full vaccination status of children was low compared with other findings in the country. Variables such as education of women, birth weight of children, attending ANC, giving birth at a health facility and wealth status were factors associated with access and the continuum of childhood vaccination. Therefore, enhancing women’s education and strengthening maternal healthcare services are of paramount importance for improving childhood vaccination status. Furthermore, the government has to design a compensation mechanism for costs relating to childhood vaccination. On top of that, researchers had better evaluate the immune status of vaccinated children and assess the vaccine cold chain management system to provide informed recommendation.

## Data Availability

The datasets used during the current study are available from the corresponding author on reasonable request.

## Abbreviations

ANC: Ante-Natal Care
AOR: Adjusted Odds Ratio
BCG: Bacilli Calamite Guerin
DPT: Diphtheria, Pertussis, and Tetanus
EA: Enumeration Area
EDHS: Ethiopia Demography Health Survey
EPI: Expanded program of immunization
IQR: Interquartile range
OPV: Oral Polio Vaccine
OR: Odds Ratio
PCV: Pneumococcal Conjugate Vaccine
PHC: Population and Housing Census
PNC: Post-Natal Care
SD: Standard Deviation
USD: United States Dollar
VPD: Vaccine-Preventable Diseases
WHO: World Health Organization
